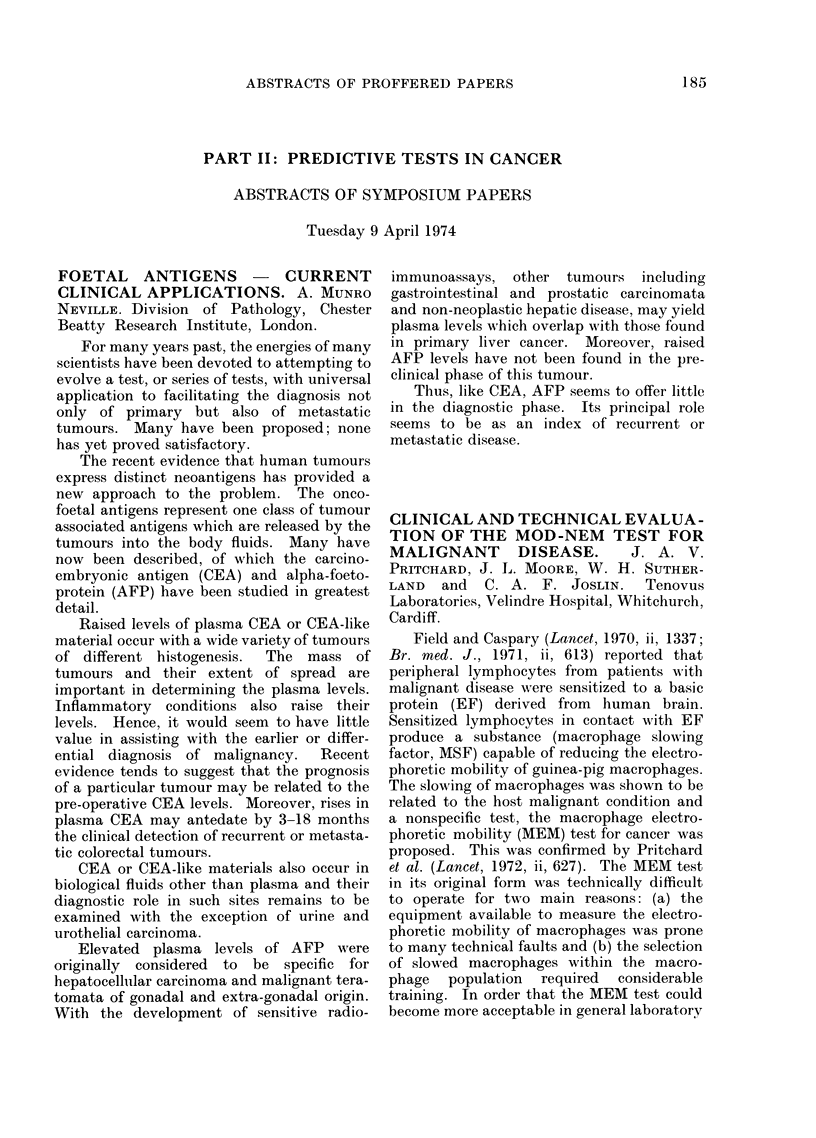# Proceedings: Foetal antigens--current clinical applications.

**DOI:** 10.1038/bjc.1974.172

**Published:** 1974-08

**Authors:** A. M. Neville


### PART II: PREDICTIVE TESTS IN CANCER

### ABSTRACTS OF SYMPOSIUM PAPERS

Tuesday 9 April 1974

**FOETAL ANTIGENS — CURRENT CLINICAL APPLICATIONS.** A. MUNRO NEVILLE. Division of Pathology, Chester Beatty Research Institute, London.

For many years past, the energies of many scientists have been devoted to attempting to evolve a test, or series of tests, with universal application to facilitating the diagnosis not only of primary but also of metastatic tumours. Many have been proposed; none has yet proved satisfactory.

The recent evidence that human tumours express distinct neoantigens has provided a new approach to the problem. The onco-foetal antigens represent one class of tumour associated antigens which are released by the tumours into the body fluids. Many have now been described, of which the carcino-embryonic antigen (CEA) and alpha-foeto-protein (AFP) have been studied in greatest detail.

Raised levels of plasma CEA or CEA-like material occur with a wide variety of tumours of different histogenesis. The mass of tumours and their extent of spread are important in determining the plasma levels. Inflammatory conditions also raise their levels. Hence, it would seem to have little value in assisting with the earlier or differential diagnosis of malignancy. Recent evidence tends to suggest that the prognosis of a particular tumour may be related to the pre-operative CEA levels. Moreover, rises in plasma CEA may antedate by 3–18 months the clinical detection of recurrent or metastatic colorectal tumours.

CEA or CEA-like materials also occur in biological fluids other than plasma and their diagnostic role in such sites remains to be examined with the exception of urine and urothelial carcinoma.

Elevated plasma levels of AFP were originally considered to be specific for hepatocellular carcinoma and malignant teratomata of gonadal and extra-gonadal origin. With the development of sensitive radioimmunoassays, other tumours including gastrointestinal and prostatic carcinomata and non-neoplastic hepatic disease, may yield plasma levels which overlap with those found in primary liver cancer. Moreover, raised AFP levels have not been found in the preclinical phase of this tumour.

Thus, like CEA, AFP seems to offer little in the diagnostic phase. Its principal role seems to be as an index of recurrent or metastatic disease.